# Methylmalonic acid promotes colorectal cancer progression via activation of Wnt/β-catenin pathway mediated epithelial–mesenchymal transition

**DOI:** 10.1186/s12935-023-02973-z

**Published:** 2023-07-05

**Authors:** Chunhua Hu, Mujie Ye, Jianan Bai, Pengfei Liu, Feiyu Lu, Jinhao Chen, Ping Yu, Tiaotiao Chen, Xiaoting Shi, Qiyun Tang

**Affiliations:** 1 Department of Geriatric Gastroenterology, Neuroendocrine Tumor Center, Jiangsu Province Hospital, The First Affiliated Hospital of Nanjing Medical University, Institute of Neuroendocrine Tumor, Nanjing Medical University, NO. 300 Guangzhou Road, Nanjing, China; 2 Department of Gastroenterology, Jiangyin People’s Hospital, Jiangyin, Jiangsu Province China

**Keywords:** Colorectal cancer, Age, Methylmalonic acid, Epithelial–mesenchymal transition, Wnt, β-catenin

## Abstract

**Background:**

It has been manifested in several studies that age-related metabolic reprogramming is associated with tumor progression, in particular, colorectal cancer (CRC). Here we investigated the role of upregulated metabolites of the aged serum, including methylmalonic acid (MMA), phosphoenolpyruvate (PEP), and quinolinate (QA), in CRC.

**Methods:**

Functional assays including CCK-8, EdU, colony formation and transwell experiments were used to ascertain which upregulated metabolite of elderly serum was related to tumor progression. RNA-seq analysis was conducted to explore the potential mechanisms of MMA-induced CRC progression. Subcutaneous tumorigenesis and metastatic tumor models were constructed to verify the function of MMA in vivo.

**Results:**

Among three consistently increased metabolites of the aged sera, MMA was responsible for tumorigenesis and metastasis in CRC, according to functional assays. The promotion of Epithelial–mesenchymal transition (EMT) was observed in CRC cells treated with MMA, on the basis of protein expression of EMT markers. Moreover, combined with transcriptome sequencing, Wnt/β-catenin signaling pathway was activated in CRC cells treated with MMA, which was verified by western blot and qPCR experiments. Furthermore, animal assays demonstrated the pro-proliferation and promotion of metastasis role of MMA in vivo.

**Conclusion:**

We have identified that age-dependent upregulation of MMA in serum promoted the progression of CRC via Wnt/β-catenin signaling pathway mediated EMT. These collective findings provide valuable insights into the vital role of age-related metabolic reprogramming in CRC progression and propose a potential therapeutic target for elderly CRC.

**Supplementary Information:**

The online version contains supplementary material available at 10.1186/s12935-023-02973-z.

## Introduction

Colorectal cancer (CRC) is the third most common malignancy in global incidence and the second in cancer-related mortality. According to GLOBOCAN 2020 statistics, the incidence and mortality rates of CRC in 2020 were 10.0% and 9.4%, respectively [1]. Metastasis is the critical cause of CRC-related death [2], and the liver is the most common site of metastasis in CRC [3]. The median survival time of liver metastasis is generally no more than 12 months, remaining a huge challenge for CRC therapy [4]. Therefore, it is necessary to elucidate the underlying mechanisms of CRC metastasis to improve clinical prognosis.

Age is the single most important and unalterable risk factor for cancer. The incidence rate of CRC escalates rapidly as age increases. Due to the aggravation of population aging, the incidence of cancer in the elderly is increasing [5]. The risk of cancer incidence rate and related mortality among people over 65 years of age is greatly increased [6, 7]. More than half of CRC patients were older than 65 years at the time of diagnosis and the mortality rate of this group is about 68% [8]. Moreover, elderly patients with cancers tend to progress rapidly with multiple metastases and poor prognosis. Age seems to play a key role in tumorigenesis and metastasis.

Several studies have reported the complex relationship between age and tumor metastasis and progression [9]. Knudsen’s hypothesis states that the frequency of cancer will be higher with age because it needs time for cells to accumulate sufficient genetic mutations to make them exceed a certain mutation threshold and enter a comprehensive carcinogenesis process [10]. This is the most common explanation of age-related cancer. However, the effects of diet and physical exercise on metabolic aging were unveiled [11]. More and more studies focused on the influence of diet, exercise and lifestyle on tumor progression and metastases [12–14].

Metabolic reprogramming is a well-recognized hallmark of cancer progression, especially the process of tumor metastasis, because tumor is also a metabolic disease. On the one hand, the process of tumor metastasis requires a large amount of energy supply through energy metabolism. On the other hand, some certain metabolites are known to be onco-metabolites that promote tumor progression and metastasis. Gomes et al. showed that aged-donor serum promoted tumor progression. Methylmalonic acid (MMA), phosphoenolpyruvate (PEP), and quinolinate (QA) were identified as consistently increased metabolites of the aged sera through targeted metabolomics [15]. Moreover, Solvang's study revealed that QA increased with age and was strongly associated with mortality and frailty, particularly in persons ≥ 70 years [16].

The aim of the present study was to explore the associations between age and serum metabolic changes in CRC patients. In terms of understanding the complex relationship between age and metabolites on the progression of CRC and clarifying the underlying molecular mechanism, HCT116 and SW480 cells were treated with MMA, PEP and QA, respectively. The effects of three metabolites on CRC cells were assessed by proliferation, migration, invasion, and EMT, which is a hallmark of migratory and invasive cells, and metastasis in cancer cells [17, 18].

## Materials and methods

### Cell lines and cell culture treatments

Human CRC cells HCT116 and SW480 were purchased from Cell Bank of Chinese Academy of Sciences in Shanghai. HCT116 cells were cultured in RPMI-1640 medium (Gibco, Carlsbad, CA, USA) supplemented with 10% fetal bovine serum (FBS, Yeasen Biotechnology, Shanghai, China) and 1% penicillin–streptomycin (Gibco). SW480 cells were cultured in L15 medium (Fuheng, Shanghai, China) supplemented with 10% FBS and 1% penicillin–streptomycin. All cell lines were maintained in a thermostatic incubator with 5% CO_2_ at 37 ℃.

To explore the effects of age on the progression of CRC, HCT116 and SW480 cells were plated in normal culture media and the next day treated with the top 3 upregulated metabolites in the aged serum, including 5 mM QA (Sigma-Aldrich), 5 mM PEP (Sigma-Aldrich), 5 mM MMA (Sigma-Aldrich), or vehicle (0.1% DMSO for QA; double distilled water for MMA and PEP). For all acidic treatments, 25 mM HEPES (Sigma-Aldrich) was added to the treatment media to buffer potential changes in pH, and the media were replaced every day during the treatments.

### Quantitative real-time PCR assay

Total RNA was extracted with TRIzol reagent (Life, USA) and reverse transcribed to cDNA using a specific cDNA synthesis kit (Yeasen). SYBR green master mix (Yeasen) was used for quantitative PCR assay (Roche). The determination conditions are as follows: pre-denaturation at 95℃ for 5 min, followed by 35 cycles of denaturation at 95℃ for 30 s, annealing at 58℃ for 30 s, and extension at 72℃ for 30 s. The gene expression results were normalized to GAPDH and analyzed using GraphPad Prism 6 software. The specific primers were shown in Additional file 1: Table S1.

### Western blot assay

Total proteins were extracted from cultured cells or tumor tissue using NP40 lysis buffer containing 1% 100 mM phenylmethanesulfonyl fluoride (Beyotime, Nantong, China). Samples were separated by 10% SDS-PAGE and transferred onto nitrocellulose (NC) filter membrane (Millipore, USA). Membranes were blocked with Tris-Buffered Saline Tween-20 buffer (TBST) containing 8% defatted milk for 1–2 h and incubated with the corresponding primary antibody (listed in Additional file 1: Table S2) at 4 ℃ overnight. The membranes were washed with TBST for three times, and then incubated with the appropriate secondary antibodies for 1 h at room temperature. After washing with TBST for three times, the membranes were exposed to enhanced chemiluminescence substrate detection solution (NCM Biotech, Suzhou, China). Image J software was used to quantify the gray value of the bands.

### Cell proliferation assay

To assess the proliferation of HCT116 and SW480 cells, cell counting kit-8 (CCK-8), 5-ethynyl-2′-deoxyuridine (EdU), and colony formation assay were performed. CCK-8 assay was executed according to the manufacturer’s recommendations. About 5 × 10^3^ cells/well of HCT116 and SW480 cells were seeded in 96-well plates and incubated for 0, 24, 48, and 72 h, followed by the addition of 10 ul CCK8 reagent (Yeasen) to each well and incubated for 2 h in a constant temperature incubator. After that, the optical cell density was detected in 450 nm microplate reader (Thermo Fisher, USA).

For EDU assay, cells were treated with 50 μM EDU (1:1000, RiboBo, Guangzhou, China) medium at 37 ℃ for 2 h, then fixed with 4% paraformaldehyde for 30 min. After permeabilized with 0.5% Triton-X, the cells were successively reacted with 1 × Apollo dyeing reaction solution and Hoechst 33,342 (RiboBo) for 30 min. Finally, images were visualized using fluorescence microscope and analyzed by Image J and GraphPad Prism 6 software.

For the colony formation assay, 3 × 10^3^ cells were seeded in each 6-well plate cultured with normal medium for 14 days in a constant temperature incubator. The cells were fixed with 4% paraformaldehyde for 30 min and then stained with 0.25% crystal violet for 30 min.

### Migration and invasion assays

Transwell chambers (Corning, USA) with 8 μm micropore were used. 2 × 10^5^ cells/ well for the cell migration experiments and 4 × 10^5^ cells/ well with a layer of matrigel (Becton, Dickinson) covering for the cell invasion experiments, were separately seeded into upper wells without FBS, whereas the medium in the bottom section was supplemented with 30% FBS. After incubation for 48 h, the cells that migrated or invaded to the lower chamber were fixed with 4% paraformaldehyde for 30 min and stained with 0.25% crystal violet for 30 min.

### RNA-seq and analysis

Total RNA was obtained from HCT116 cells treated with 5 mM MMA for 10 days and control groups with TRIzol^®^ (Takara Bio, Inc.). Total RNA was sent to Hangzhou Lianchuan Biotechnology Co., Ltd for further processing and RNA-seq analysis. Briefly, Illumina Paired End Sample Prep kits (Illumina, Inc.) were used to prepare libraries. Each cDNA library was sequenced using an Illumina Hiseq 4000 (cat. no. PE150; Illumina, Inc.). Differential expression levels of mRNA transcripts between the MMA-treated and control groups were measured and subsequent GO and KEGG analysis were performed.

### Animal assays

Male athymic nude mice (4–6 weeks of age) were bred in laminar flow hoods using plastic cages with filter caps. The mice were maintained in compliance with Nanjing Medical University Institutional Animal Care and Use Committee (IACUC) protocols. The tumor size limit on the protocol was 15 mm on the largest dimension or 2.5 cm^3^ tumor volume or 10% of body weight, whichever was reached first. To achieve increased circulatory MMA concentrations and ascertain the effect of MMA on tumor growth in vivo, mice were treated with MMA ((200 μg MMA/g/day) through the drinking water.

For the in vivo xenograft model, a total of 1 × 10^6^ HCT116 cells (treated with MMA or ddH_2_O for 10 days) were suspended in 100 μl PBS and subcutaneously injected into the axillary region of the mice. Four weeks after cell injection, the mice were anesthetized by inhaling carbon dioxide and sacrificed by cervical dislocation, and the volume and weight of each excised subcutaneous tumor were measured. The tumor volumes were calculated by the following formula: tumor volume (mm^3^) = 0.5 × length × width^2^. Besides, tumor tissues were fixed and sectioned for immunohistochemistry of Ki67, E-cadherin and N-cadherin.

For secondary organ colonization assay in mice, 1 × 10^5^ mCherry- luciferase HCT116 cells (treated with MMA or ddH_2_O for 10 days) were suspended in 100 μl PBS and injected into the tail vein. Metastases were monitored using IVIS Spectrum CT Pre-Clinical In Vivo Imaging System (Perkin-Elmer), and luminescence was measured and quantified to determine secondary organ colonization after 7 weeks.

### Statistical analysis

Data analyses were performed using GraphPad Prism 6.0 software. Data are represented as mean ± SD of individual data points of at least three independent samples. Two-tailed student’s *t*-test was used to assess significance. In all types of statistical analysis values of p < 0.05 were considered significant.

## Results

### MMA promotes cell proliferation in CRC cells

**Fig. 1:**
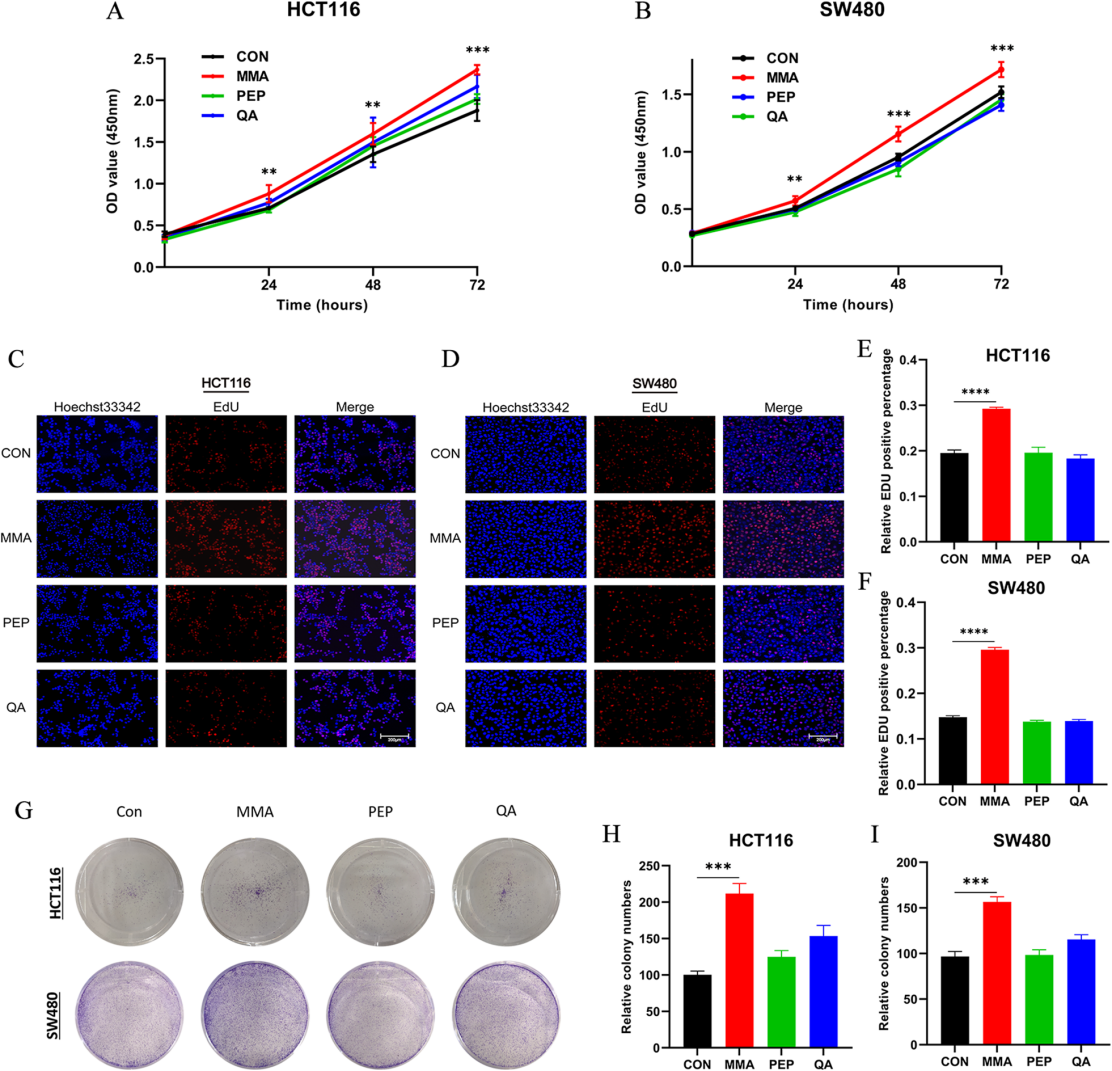
MMA promotes cell proliferation in CRC cells. **A**, **B** CCK-8 assay showed that MMA was the most significant metabolite promoting the proliferation of both HCT116 (**A**) and SW480 (**B**) cells. **C**–**F** EdU assay indicated that MMA was significant for the increasing proliferation in both HCT116 (**C**, **E**) and SW480 (**D**, **F**) cells. Magnification: × 200. **G**–**I** Colony formation assay indicated that cell colony formation was increased in both HCT116 (**G**, **H**) and SW480 (**G**, **I**) cells. (**P < 0.01, ***P < 0.001, ****P < 0.0001)

In order to ascertain the effect of serum metabolic changes of the elderly on CRC, we treated HCT116 and SW480 cells with three metabolites respectively, including MMA, PEP and QA, which were significantly elevated in the old serum. The results of the CCK-8 assay showed that MMA was the most significant metabolite promoting the capacity of both HCT116 and SW480 cells to proliferate (Fig. 1A, B). To further investigate the promoting influence of age-induced circulatory factors on cell proliferation, the EdU assay was performed in two CRC cell lines. The results indicated that only MMA was significant for the increasing capacity of proliferation in CRC cells (Fig. 1C–F). Furthermore, the colony formation assays revealed that MMA was the most significant serum metabolite promoting cell colony formation (Fig. 1G–I). Taken together, these results demonstrated that among the top three significant up-regulated metabolites of the old sera, only MMA was responsible for promoting cell proliferation in CRC cells.

### MMA promotes cell migration, invasion and regulates EMT in CRC cells

**Fig. 2:**
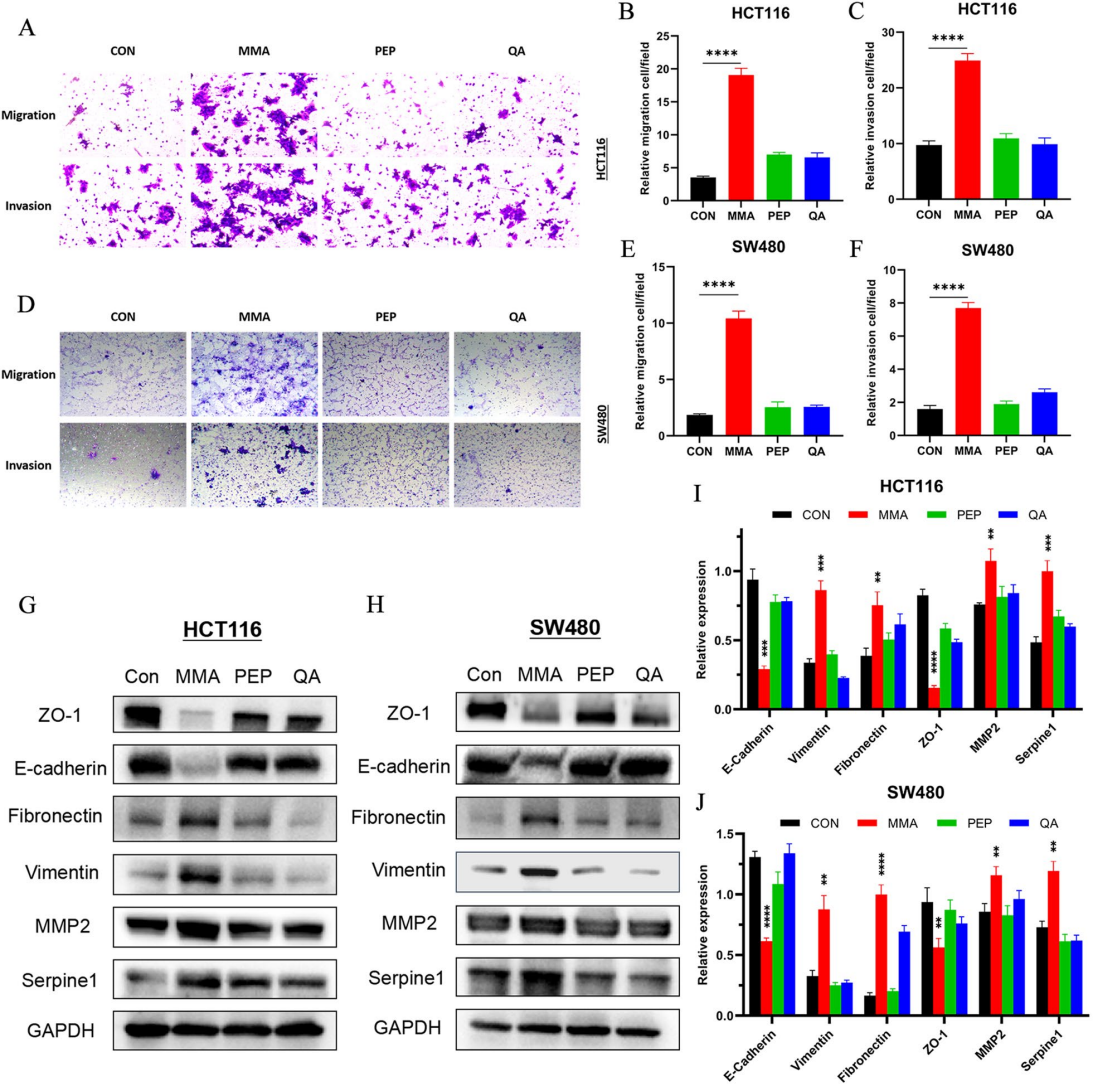
MMA promotes cell migration, invasion and regulates EMT in CRC cells. **A**–**F** Transwell assays indicated that MMA promoted the migration and invasion of both HCT116 (**A**–**C**) and SW480 (**D**–**F**) cells most remarkably. (**G**–**J**) Western blot analysis of alterations in protein levels of EMT -related markers showed that epithelial markers were downregulated and mesenchymal markers were upregulated in both HCT116 (G, I) and SW480 (H, J) cells treated with MMA. (**P < 0.01, ***P < 0.001, ****P < 0.0001)

To further investigate the pro-aggressive effects of age-induced serum metabolic changes on migration and invasion of CRC cells, transwell assays were performed. It showed that MMA was the most remarkable serum metabolite to facilitate cell migration and invasion in both HCT116 and SW480 cells (Fig. 2A–F). EMT is a key process in cancer cell metastasis. According to the analysis of cellular phenotypes, EMT promotion may be involved in the pro-aggressive effects of MMA on CRC cells. Western blot was used to analyze EMT markers induced by the three metabolites in HCT116 and SW480 cells. It showed that epithelial markers, including E-cadherin and ZO-1, were remarkably downregulated only in MMA-induced cells both in HCT116 and SW480 cell lines. Meanwhile, the mesenchymal markers, including fibronectin and vimentin, was upregulated by MMA in the two cell lines. Consistent with these findings, the expression of aggressive markers,including MMP2 and Serpine1, increased significantly in MMA-induced cells compared to its expression in control cells (Fig. 2G–J). These results suggested that MMA promoted EMT-mediated migration and invasion in CRC cells.

### MMA drives EMT in CRC cells through activation of Wnt/β-catenin signaling

**Fig. 3:**
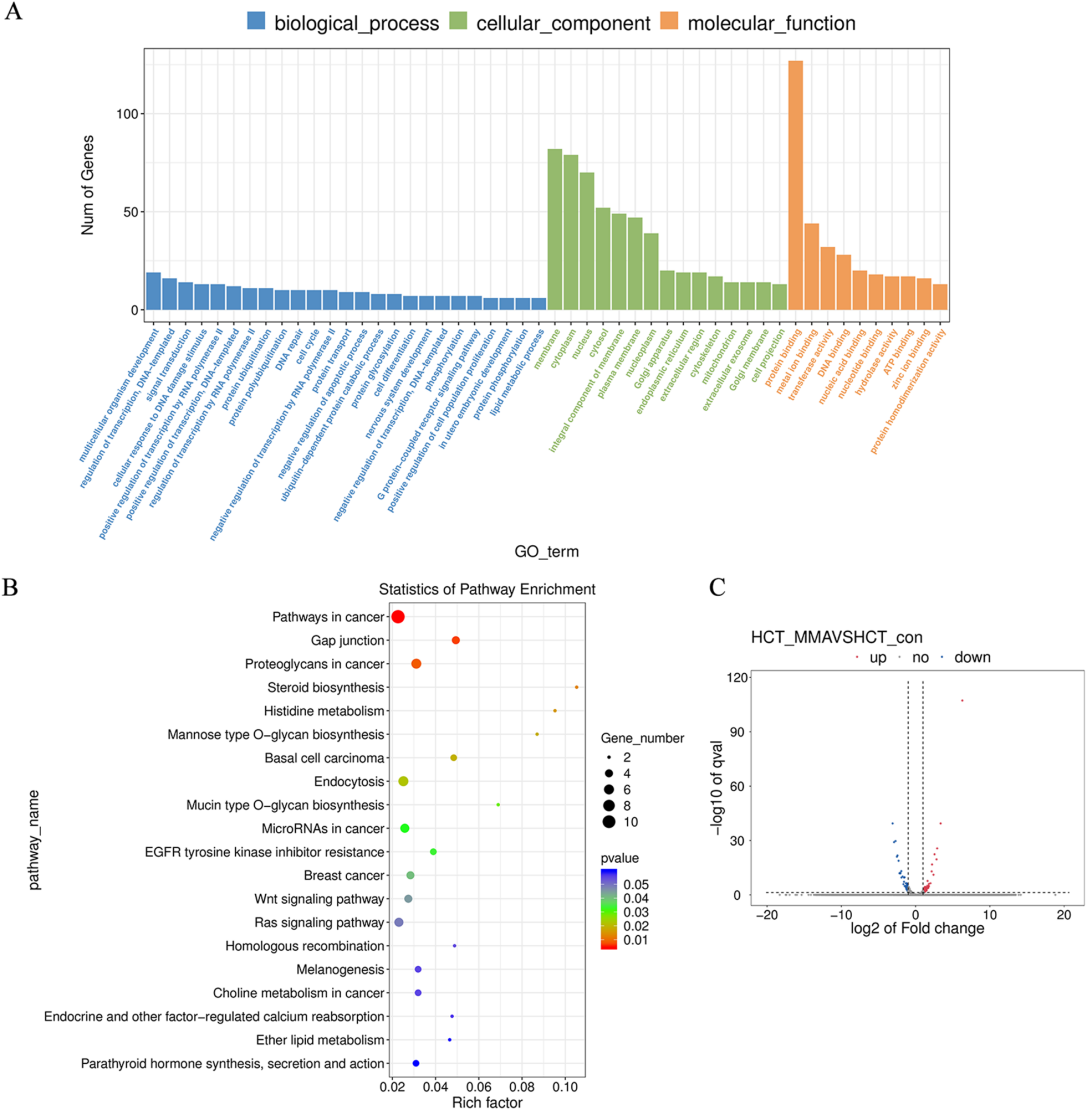
Transcriptomic analysis in HCT116 cells treated with MMA. **A** Gene Ontology (GO) analysis showed that MMA positively regulated cell population proliferation in HCT116 cells treated with MMA. **B** KEGG pathway enrichment analysis revealed that Wnt signaling pathway may be involved in MMA-induced HCT116 cells. **C** The volcano plot visualized the genes that were significantly changed ≥ 1.5 fold in HCT116 cells treated with MMA

**Fig. 4:**
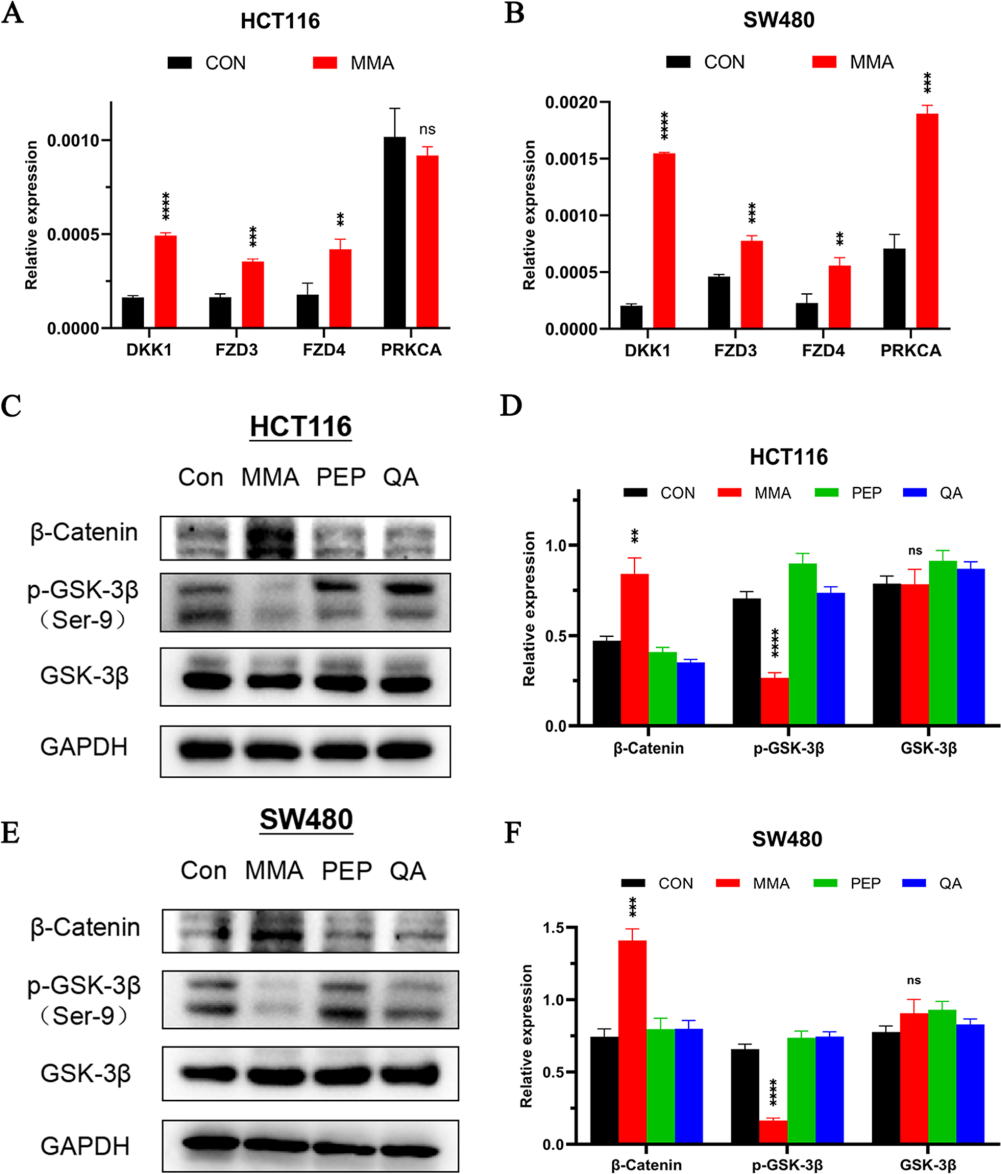
MMA drives EMT in CRC cells through Wnt/β-catenin signaling. **A**, **B** Wnt signaling pathway related genes, including FZD3, DKK1, PRKCA and FZD4, were upregulated in both HCT116 (**A**) and SW480 (**B**) cells treated with MMA, as observed with gene set enrichment analysis (GSEA) and RT-PCR. **C**–**F** Western blot analysis of the protein levels of Wnt/β-catenin signaling -related proteins showed that the expression of β-catenin increased while p-GSK-3β (Ser9) decreased in both HCT116 (**C**, **D**) and SW480 (**E**, **F**) cells treated with MMA. (**P < 0.01, ***P < 0.001, ****P < 0.0001)

To demonstrate how MMA promoted EMT-mediated migration and invasion in CRC cells, we conducted a global transcriptomic analysis in HCT116 cells treated with MMA for 10 days. Gene Ontology (GO) analysis showed that MMA positively regulated cell population proliferation (Fig. 3A), which was consistent with the cellular phenotypes above. KEGG pathway enrichment analysis revealed that the Wnt signaling pathway might be involved in MMA-induced cells (Fig. 3B). A volcano plot visualizing the genes that were significantly changed ≥ 1.5 fold was plotted (Fig. 3C). Furthermore, four genes related to Wnt pathway were significantly upregulated according to the gene set enrichment analysis (GSEA), including FZD3, DKK1, PRKCA and FZD4, which was validated by qPCR assay (Additional file 2: Table S3, Fig. 4A, B). It is well-known that Wnt signaling is a common upstream signal of EMT [19]. To further clarify whether MMA regulated Wnt signaling pathway, we verified the expression of the key point β-catenin and downstream protein p-GSK-3β by western blot assay, and the results showed that β-catenin increased while p-GSK-3β (Ser9) decreased only in MMA induced CRC cells (Fig. 4C–F). These results prompted that MMA facilitated the aggressivity of CRC cells by inducing EMT through Wnt/β-catenin signaling pathway.

### MMA accelerates tumor growth and metastasis in mice

**Fig. 5:**
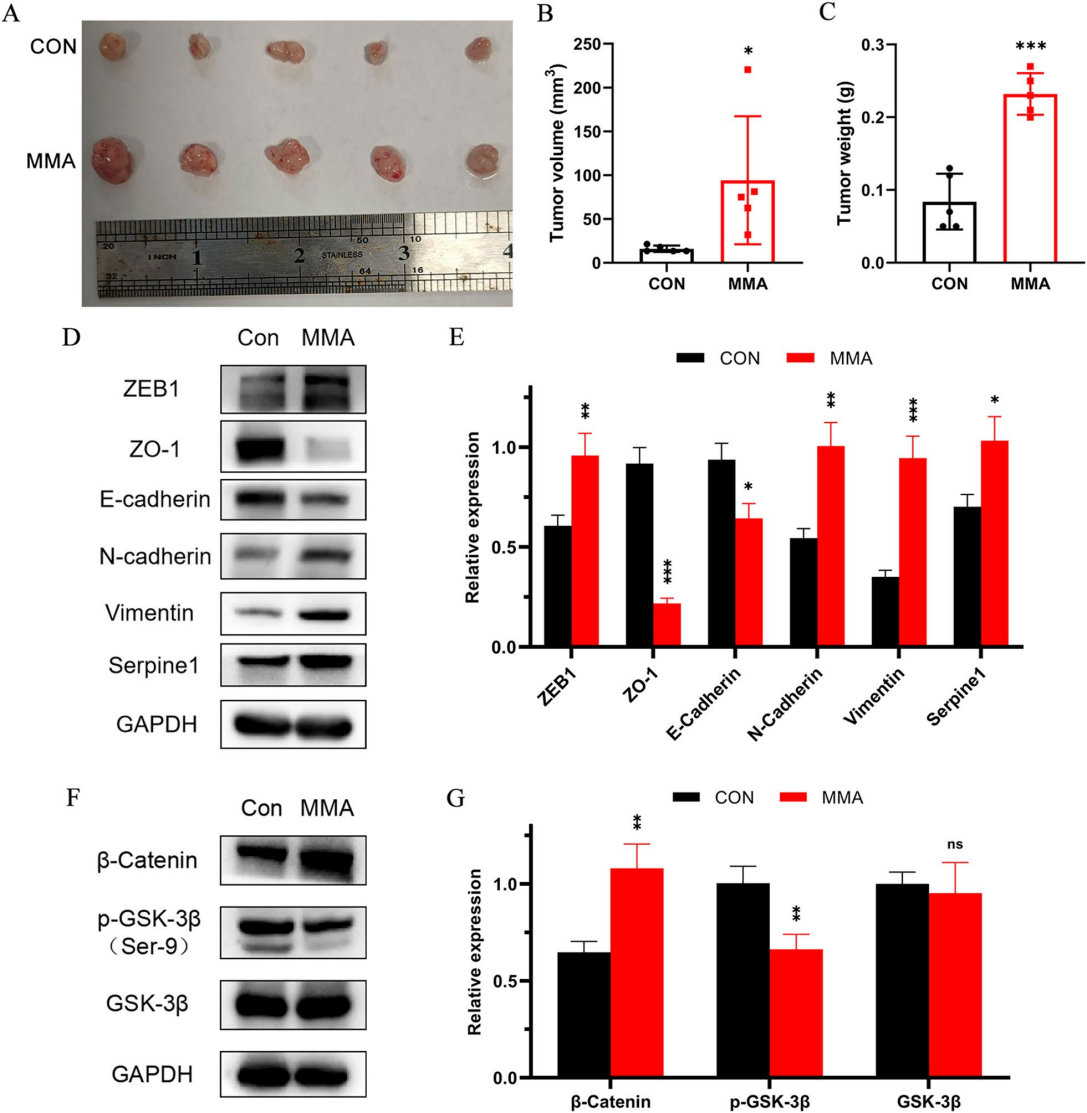
MMA promotes tumor growth in vivo. **A** The images of primary tumor samples obtained from mice subcutaneously injected with HCT116 cells treated with MMA and control cell groups. **B**, **C** The relative tumor volumes (**B**) and weights (**C**) at the endpoint were analyzed (n = 5). **D**, **E** Western blot analysis of the tumor samples of EMT-related proteins showed that epithelial markers were downregulated and mesenchymal markers were upregulated in the MMA-induced group. **F**, **G** Western blot analysis of the tumor samples of Wnt/β-catenin signaling-related proteins showed that the expression of β-catenin increased while p-GSK-3β (Ser9) decreased in the MMA-induced group. (*P < 0.05, **P < 0.01, ***P < 0.001)

**Fig. 6:**
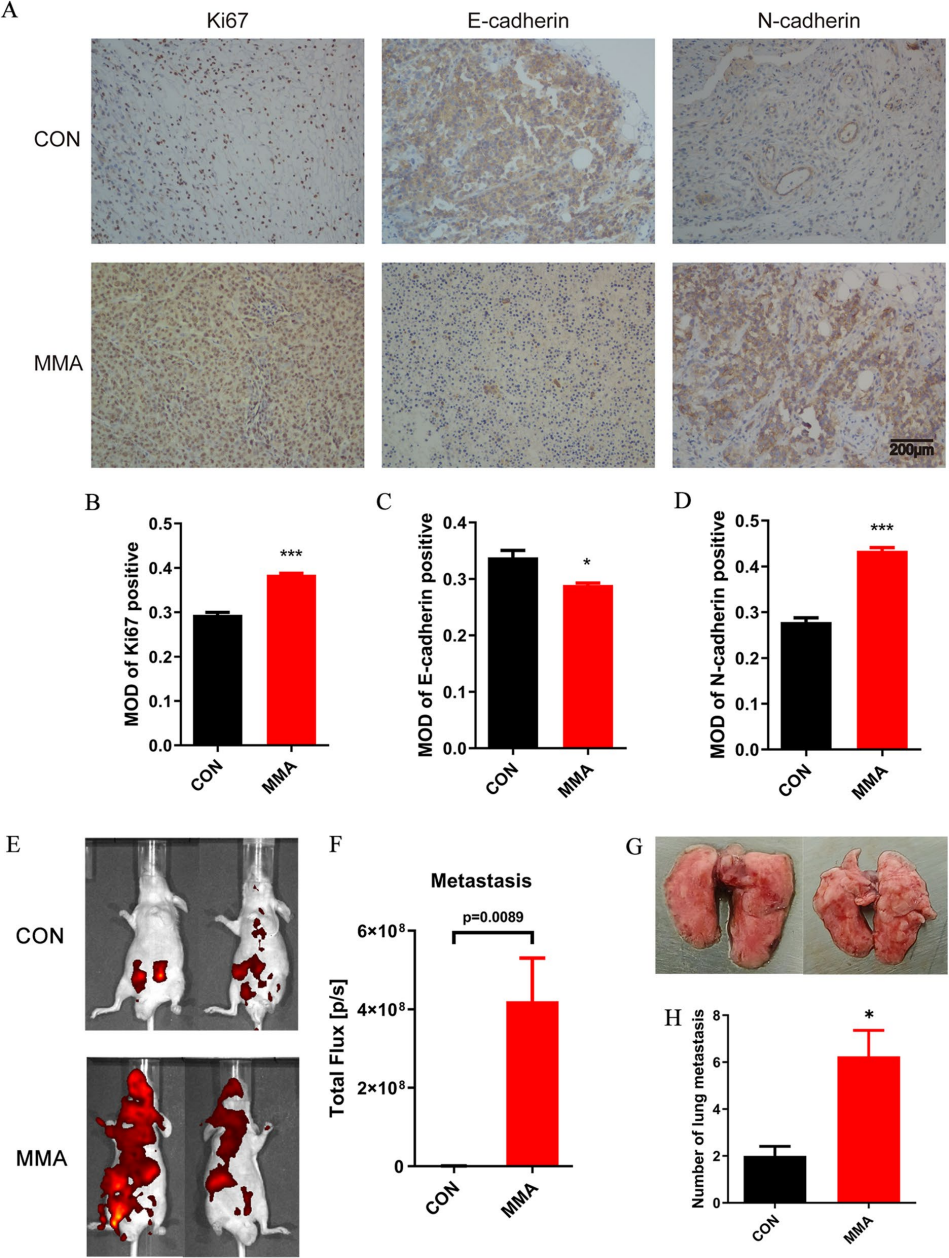
MMA promotes tumor metastasis in vivo. **A–D** Representative IHC images showing expression of Ki67 (**B**), E-cadherin (**C**) and N-cadherin (**D**) in subcutaneous tumor tissues. **E**, **F** Bioluminescence intensity of the metastases in mice that were injected through the tail vein with mCherry- luciferase HCT116 cells (treated with MMA or ddH_2_O for 10 days). **G**, **H** Representative images of metastatic lung tumors (**G**) and the number of lung tumors were quantitatively analyzed (**H**). (*P < 0.05, ***P < 0.001)

Subcutaneous tumorigenesis model was used to assess the effects of MMA on the capacity of tumourigenicity in CRC in vivo. HCT116 cells were treated with MMA for 10 days prior to injection into the nude mice, and the volume and weight of each excised subcutaneous tumor were measured after four weeks. The results showed that MMA accelerated tumor xenograft growth in vivo (Fig. 5A–C). Western blot assay of excised tumor tissue further confirmed that MMA induced a decrease in ZO-1, E-cadherin and p-GSK-3β (Ser9) expression and an increase in ZEB-1, N-cadherin, Vimentin, Serpine1 and β-catenin expression, which demonstrated that MMA induced EMT through Wnt/β-catenin signaling pathway in CRC in vivo (Fig. 5D–G). In addition, Ki67 expression levels were significantly higher in MMA-induced tumors according to the immunohistochemistry results. Furthermore, decreased E-cadherin and increased N-cadherin expression levels were shown by immunohistochemistry in situ in the MMA-induced tumors of nude mice (Fig. 6A–D). In order to assess the effect of MMA on metastatic ability in CRC in vivo, mCherry-luciferase HCT116 cells treated with MMA or ddH_2_O for 10 days were injected through the tail vein. The bioluminescence intensity of metastases in the MMA-treated mice was significantly higher than that in the control group (Fig. 6E, F). The number of lung metastases in the MMA-treated mice was significantly higher than that in the control group (Fig. 6G, H). Overall, these data supported that MMA promoted tumorigenicity and tumor metastasis in vivo.

## Discussion

Cancer and aging are inextricably interconnected, and it is widely thought that CRC is predominantly a disease of ageing. Previous studies have shown that morbidity and mortality of CRC increase rapidly after the age of 50, with an estimated 90% of cases and deaths worldwide occurring at ages over 50 years [20, 21]. Although research on aging and age-related diseases have emerged in recent years, the underlying mechanism remains poorly defined due to its complexity. With the advent of an aging population, the management of CRC in the elderly will become a global burden in the next few decades. However, little is known about the mechanisms underlying the complex relationship between age and tumor progression. Therefore, there is an increasingly urgent need for basic research that elucidate the possible mechanisms of age on tumor progression.

Despite the time length of carcinogenesis being the most obvious explanation, more and more studies have shown that the changes in serum metabolism with aging may provide an alternative approach to illustrate the mechanism of age-related colorectal cancer progression [22]. Altered metabolism is one of the hallmarks of cancer, and the metabolic change is a key factor in the ability of cancer cells to acquire metastasis [23–25]. Gomes et al. reported that among the three upregulated metabolites of aged serum, MMA might facilitate the progression of breast cancer and lung cancer through TGF-β signaling pathway [15]. These results led us to a new hypothesis that metabolic change of aged serum might promote the progression of CRCs. CRC cells were treated with MMA, PEP and QA, respectively, in order to ascertain the association between metabolic change in old and the progression of CRCs. In line with this, this study verified the effects of these three upregulated metabolites on CRC cells and uncovered that only MMA could increase the progression of CRCs, both in vivo and in vitro.

Methylmalonic acid (MMA) is a by-product of propionate metabolism, which provides fuel for the tricarboxylic acid (TCA) cycle. Methylmalonyl coenzyme A is converted to succinyl coenzyme A in a VitB12-dependent manner and combines with folic acid to convert homocysteine (HC) into methionine [26]. Gomes et al. have demonstrated that the downregulation of methylmalonyl coenzyme A epimerase resulted in the accumulation of MMA to expedite cancer cell aggressiveness in breast and lung cancer [27]. However, future studies should investigate whether this mechanism is still applicable in other tumor patients, for instance, CRC.

EMT is a process in which tumor cells lose their epithelial characteristics and transform into more aggressive mesenchymal cells, which is a vital mechanism for tumor metastasis [28]. It has been reported that EMT is regulated by multiple pathways such as Wnt, TGF-β, MAPK and PI3K [29–33]. There have been a lot of studies that indicated the abnormality of Wnt/β-catenin pathway was related to various human tumors, most notably CRCs. In this study, it was Wnt signaling pathway mediated EMT that regulated the progression of CRC according to KEGG pathway enrichment analysis. There are three branches of Wnt signaling pathways: Canonical Wnt signaling pathway, that is, Wnt/β-catenin pathway, Wnt-PCP pathway and Wnt-Ca^2+^ signaling. Current research on Wnt pathway mainly refers to Wnt/β-catenin, which is related to many diseases [34]. The accumulation of β-catenin is an important hallmark of Wnt signaling pathway activation, which is related to cell proliferation, migration and differentiation. All these results validated that MMA might motivate CRC development by activating Wnt/β-catenin signaling pathway mediated EMT. Further studies are needed to elucidate how MMA activates Wnt/β-catenin pathway in CRCs.

In summary, the present study displayed that MMA, among the three upregulated metabolites in aged serum, seems to be associated with the progression of cancer cells in elderly CRC. Wnt/β-catenin signaling pathway mediated EMT was activated in CRC cells treated with MMA. We propose that MMA accumulation in elderly serum might increase CRC metastasis through Wnt/β-catenin signaling pathway mediated EMT. These explorative results provide potential mechanisms that how age-related metabolic changes promote the aggressiveness of cancer cells in CRCs and propose a promising therapeutic target for elderly CRC.

However, some limitations are worth noting. First, Since little is known about MMA as a metabolite, further studies are needed to reveal the mechanism of its functional effects. The specific mechanism of how MMA promotes the activation of Wnt/β-catenin in CRC needs much further research. Second, subsequent research about MMA by Gomes et al. have shown that serum MMA not only induces EMT in tumor cells to promote tumor metastasis, but also acts on cancer-associated fibroblast (CAF) in the tumor microenvironment [34]. MMA induces ROS production in CAFs, which in turn activates various signaling pathways in CAFs and tumor cells, thus promoting tumor metastasis. It would be valuable to investigate whether MMA impacts other cells in the tumor microenvironment of CRC, such as CAFs and macrophages. Third, in order to make this discovery clinically relevant, future work should therefore be needed to find a target that inhibited the pro-metastatic effects of MMA.

## Supplementary Information

**Additional file 1: Table S1.** Primer sequence.** Table S2.** Antibody information.

**Additional file 2: Table S3.** Differentially expressed genes of HCT116 cells treated with vehicle and 5mM MMA for 10 days.

## Data Availability

All of the data of this study are available from the corresponding author.

## Abbreviations

CRC: Colorectal cancer
MMA: Methylmalonic acid
PEP: Phosphoenolpyruvate
QA: Quinolinate
EMT: Epithelial–mesenchymal transition
CCK8: Cell counting kit-8
EdU: 5-Ethynyl-2′-deoxyuridine
RT-qPCR: Quantitative real-time polymerase chain reaction
FBS: Fetal bovine serum
GAPDH: Glyceraldehyde-3-phosphate dehydrogenase
