# Supplementary material for: Methylmalonic acid promotes colorectal cancer progression via activation of Wnt/β-catenin pathway mediated epithelial–mesenchymal transition
Source: Cancer Cell Int. 2023 Jul 5;23:131. doi: 10.1186/s12935-023-02973-z (PMC10320877; doi:10.1186/s12935-023-02973-z)
Supplement: Supplementary file 1 — Additional file 1: Table S1. Primer sequence. Table S2. Antibody information. [file 12935_2023_2973_MOESM1_ESM.docx]

Additional file 1: Materials

**Table S1 Primer sequence**

| Gene | Sequence |
| --- | --- |
| DKK1 for qRT-PCR | F: 5′- CCTTGAACTCGGTTCTCAATTCC -3′ |
|  | R: 5′- CAATGGTCTGGTACTTATTCCCG -3′ |
| FZD3 for qRT-PCR | F: 5′- GTTCATGGGGCATATAGGTGG -3′ |
|  | R: 5′- GCTGCTGTCTGTTGGTCATAA -3′ |
| FZD4 for qRT-PCR | F: 5′- CCTCGGCTACAACGTGACC -3′ |
|  | R: 5′- TGCACATTGGCACATAAACAGA -3′ |
| PRKCA for qRT-PCR | F: 5′- GATAAACTCGGAATCCCGACTG -3′ |
|  | R: 5′- TCCTCCAATGCTGATCCCGAT -3′ |
| GAPDH for qRT-PCR | F: 5′- GGAGCGAGATCCCTCCAAAAT -3′ |
|  | R: 5′- GGCTGTTGTCATACTTCTCATGG -3′ |

**Table S2 Antibody information**

| Antibody | Vendor | Catalog number |
| --- | --- | --- |
| E-cadherin | CST | 3195T |
| Vimentin | CST | 5741T |
| Fibronectin | Abcam | ab268021 |
| ZO-1 | CST | 8193T |
| MMP2 | Abcam | ab92536 |
| Serpine1 | Santa Cruz | sc-5297 |
| β-catenin | CST | 8480T |
| p-GSK-3β (ser-9) | CST | 5558T |
| GSK-3β | CST | 12456T |
| ZEB1 | Proteintech | 21544-1-AP |
| N-cadherin | Proteintech | 22018-1-AP |
| GAPDH | Proteintech | 60004-1-Ig |

Antibodies were diluted according to the manufacturer’s recommendations.

**Table S3 Differentially expressed genes of HCT116 cells treated with vehicle and 5mM MMA for 10 days**

(Please see the attachment for further information)
